# Barriers and facilitators to exclusive breastfeeding among Black mothers: A qualitative study utilizing a modified Barrier Analysis approach

**DOI:** 10.1111/mcn.13428

**Published:** 2022-09-13

**Authors:** Victoria Tran, Amelia Reese Masterson, Tomeka Frieson, Frankie Douglass, Rafael Pérez‐Escamilla, Kathleen O'Connor Duffany

**Affiliations:** ^1^ Yale School of Public Health New Haven Connecticut USA; ^2^ Community Alliance for Research and Engagement (CARE) Southern Connecticut State University and Yale School of Public Health New Haven Connecticut USA

**Keywords:** barrier analysis, breastfeeding, focus group, public health, qualitative methods, social factors, sociocultural analyses

## Abstract

Breastfeeding has health benefits for both infants and mothers, yet Black mothers and infants are less likely to receive these benefits. Despite research showing no difference in breastfeeding intentions by race or ethnicity, inequities in breastfeeding rates persist, suggesting that Black mothers face unique barriers to meeting their breastfeeding intentions. The aim of this study is to identify barriers and facilitators that Black women perceive as important determinants of exclusively breastfeeding their children for at least 3 months after birth. Utilizing a Barrier Analysis approach, we conducted six focus group discussions, hearing from Black mothers who exclusively breastfed for 3 months and those who did not. Transcripts were coded starting with a priori parent codes based on theory‐derived determinants mapped onto the Socioecological Model; themes were analysed for differences between groups. Facilitators found to be important specifically for women who exclusively breastfed for 3 months include self‐efficacy, lactation support, appropriate lactation supplies, support of mothers and partners, prior knowledge of breastfeeding, strong intention before birth and perceptions of breastfeeding as money‐saving. Barriers that arose more often among those who did not exclusively breastfeed for 3 months include inaccessible lactation support and supplies, difficulties with pumping, latching issues and perceptions of breastfeeding as time‐consuming. Lack of access to and knowledge of breastfeeding laws and policies, as well as negative cultural norms or stigma, were important barriers across groups. This study supports the use of the Socioecological Model to design multicomponent interventions to increase exclusive breastfeeding outcomes for Black women.

## Key messages

- Black mothers face unique barriers to meeting their breastfeeding intentions across the Socioecological Model. Inaccessible lactation support and supplies, the perception that breastfeeding is time‐consuming and physiological and technical issues are important barriers to breastfeeding among Black mothers in New Haven, CT. A lack of knowledge about breastfeeding laws and policies, particularly in the workplace, poses an important barrier to breastfeeding among Black women.
- Black mothers also face important facilitators to meeting their breastfeeding intentions across the Socioecological Model. Support from mothers and partners, prior breastfeeding knowledge, a strong intention to breastfeed before birth and perceiving breastfeeding as money‐saving are all important facilitators to breastfeeding among Black mothers in New Haven, CT. Multicomponent interventions guided by the Socioecological Model are needed to improve breastfeeding outcomes in Black women.

## Graphical abstract

Utilizing a Barrier Analysis approach, we conducted six focus group discussions, hearing from Black mothers who exclusively breastfed for 3 months and those who did not. Facilitators found to be important specifically for women who exclusively breastfed for 3 months include self‐efficacy, lactation support, appropriate lactation supplies, support of mothers and partners, prior knowledge of breastfeeding, strong intention before birth and perceptions of breastfeeding as money‐saving. Barriers that arose more often among those who did not exclusively breastfeed for 3 months include inaccessible lactation support and supplies, difficulties with pumping, latching issues and perceptions of breastfeeding as time‐consuming.

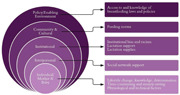

## 1 INTRODUCTION

Breastfeeding is the optimal form of nutrition for infant growth and development. Demonstrated health benefits for infants include reduced risk of asthma, obesity/overweight, hypertension, type 1 and type 2 diabetes, severe lower respiratory disease, acute otitis media, sudden infant death syndrome and gastrointestinal infections (CDC, 2020; Harder et al., 2005; Horta & Victora, 2013). There is also consistent evidence that longer breastfeeding duration is associated with improved cognitive development in children (Bernard et al., 2013; Kramer et al., 2008; Lee et al., 2016). For mothers, some of the benefits of breastfeeding include decreased risk of severe postpartum bleeding, breast and ovarian cancer, high blood pressure and type 2 diabetes (AAP, 2021; CDC, 2020).

Despite the measured benefits, there are considerable inequities in initiation, duration and exclusivity of breastfeeding between non‐Hispanic White infants and non‐Hispanic Black infants (Beauregard, 2019) and their ability to meet breastfeeding intentions (Hamner et al., 2021). According to the National Immunization Survey‐Child, Black infants had lower rates of breastfeeding initiation (69.4%) than White infants (85.9%). Black infants also had a lower rate of exclusive breastfeeding at 3 months (36.0%) than White infants (53.0%) (Beauregard, 2019). This value falls well below the Healthy People 2020 3‐month exclusive breastfeeding goal of 46.2% (HHS, 2020). These racial/ethnic disparities are also evident at the state level in Connecticut. According to the Connecticut Pregnancy Risk Assessment Monitoring System (PRAMS), in 2018, non‐Hispanic Black women had lower rates of any breastfeeding at 8 weeks (67.3%), compared to non‐Hispanic White women (74.3%) (CT DPH, 2018). In 2011, the Connecticut PRAMS showed similar disparities in exclusive breastfeeding rates at 3 months between non‐Hispanic Black infants (31.7%) and non‐Hispanic White infants (39.6%) (CT DPH, 2015), demonstrating the persistence of inequity over time.

Although wide disparities exist in engaging in breastfeeding, research has shown that intentionality to breastfeed does not vary significantly by race or ethnicity. In a study of 2070 women and children enroled in the WIC programme across 27 states, researchers found that 87.2% of non‐Hispanic Black mothers had general breastfeeding intentions compared to 86.9% of non‐Hispanic White mothers. These similarities in the rate of intent but disparities in the rate of engagement suggest that there are barriers unique to Black mothers that are affecting their ability to meet their breastfeeding intentions. One significant barrier is employment. Black women are over‐represented in the service‐sector industry, where labour protections are weaker. Thus, they have less access to adequate maternity leave or lactation breaks during the workday (DeVane‐Johnson et al., 2017). Evidence also indicates that Black women are less likely than White women to report receiving breastfeeding guidance from health care providers or counsellors (Beal et al., 2003; Kulka et al., 2011). Further, Black women are at a disproportionately higher risk of having poor birth outcomes, such as preterm birth, which can make breastfeeding more difficult (Crippa et al., 2019; Culhane & Goldenberg, 2011). Finally, Black women are more likely to experience structural racism and systemic discrimination, contributing to higher levels of stress and posttraumatic stress disorder, which can lead to lower breastfeeding rates (Giscombé & Lobel, 2005; Seng et al., 2011; Taveras et al., 2003).

To further examine these disparities, we conducted a qualitative study to explore the barriers and facilitators to exclusive breastfeeding for at least 3 months after birth among Black and African American mothers in the Greater New Haven area. New Haven, Connecticut, is a mid‐sized city in the northeastern United States with a population of approximately 134,000 people. People who identify as only non‐Hispanic Black or African American comprise about 32% of the city's population, second only to those who identify as only White (about 33% of the city's population) (United States Census Bureau, 2020). In the summer of 2020, the New Haven Breastfeeding Task Force, which works to support and conduct equitable breastfeeding work in New Haven, prioritized this study as a way to better understand breastfeeding within their community and utilize results to inform action. Conducting this study in New Haven is both a step toward continued community‐engaged research and toward increasing equity in breastfeeding throughout the state of Connecticut.

This study contributes to a growing understanding of the experiences of Black mothers that influence their breastfeeding decisions and ability to meet breastfeeding intentions. Study findings can inform public health interventions and policies to address breastfeeding disparities. Findings also support the importance of women of colour co‐designing breastfeeding interventions that aim to impact breastfeeding equity in the United States (Segura‐Pérez et al., 2021).

## 2 METHODS

### 2.1 Design and sampling

This study adapted the Barrier Analysis tool (Kittle, 2013), a rapid assessment tool that is used to identify determinants associated with a particular behaviour. It is based on two main theories of behaviour change: the Health Belief Model, which focuses on the reasons people may take action to prevent illness, and the Theory of Reasoned Action, which suggests that a person's behaviour is determined by the norms around them (Davis, 2004; Kittle, 2013). The behaviour examined was exclusive breastfeeding for at least 3 months. Participants were asked questions related to the following breastfeeding determinants: self‐efficacy, social norms, positive consequences, negative consequences, access, susceptibility/risk, severity, action efficacy, divine will, policy and culture. To evaluate differences in how the determinants affected the adoption of the behaviour, participants were recruited into two distinct focus groups: those who exclusively breastfed (EBF) for the first 3 months after birth, and those who did not exclusively breastfeed (NEBF) for the first 3 months. We adopted the Barrier Analysis tool, typically administered through surveys or individual interviews, to be used for focus groups to elicit more nuanced, in‐depth insights. Separate interview guides were created for EBFs and NEBFs to account for their potential differences in exclusive breastfeeding experiences. The adapted tool was reviewed by a Community Advisory Board (CAB) made up of community members and field experts in breastfeeding.

Eligibility criteria were mothers who were residents of the Greater New Haven area; self‐identified as Black, African American or Hispanic‐Black; gave birth within the last 18 months and had an infant who was at least 3 months old. Not all Black mothers were African American, as some were born outside the United States.

### 2.2 Data collection

**Figure 1:**
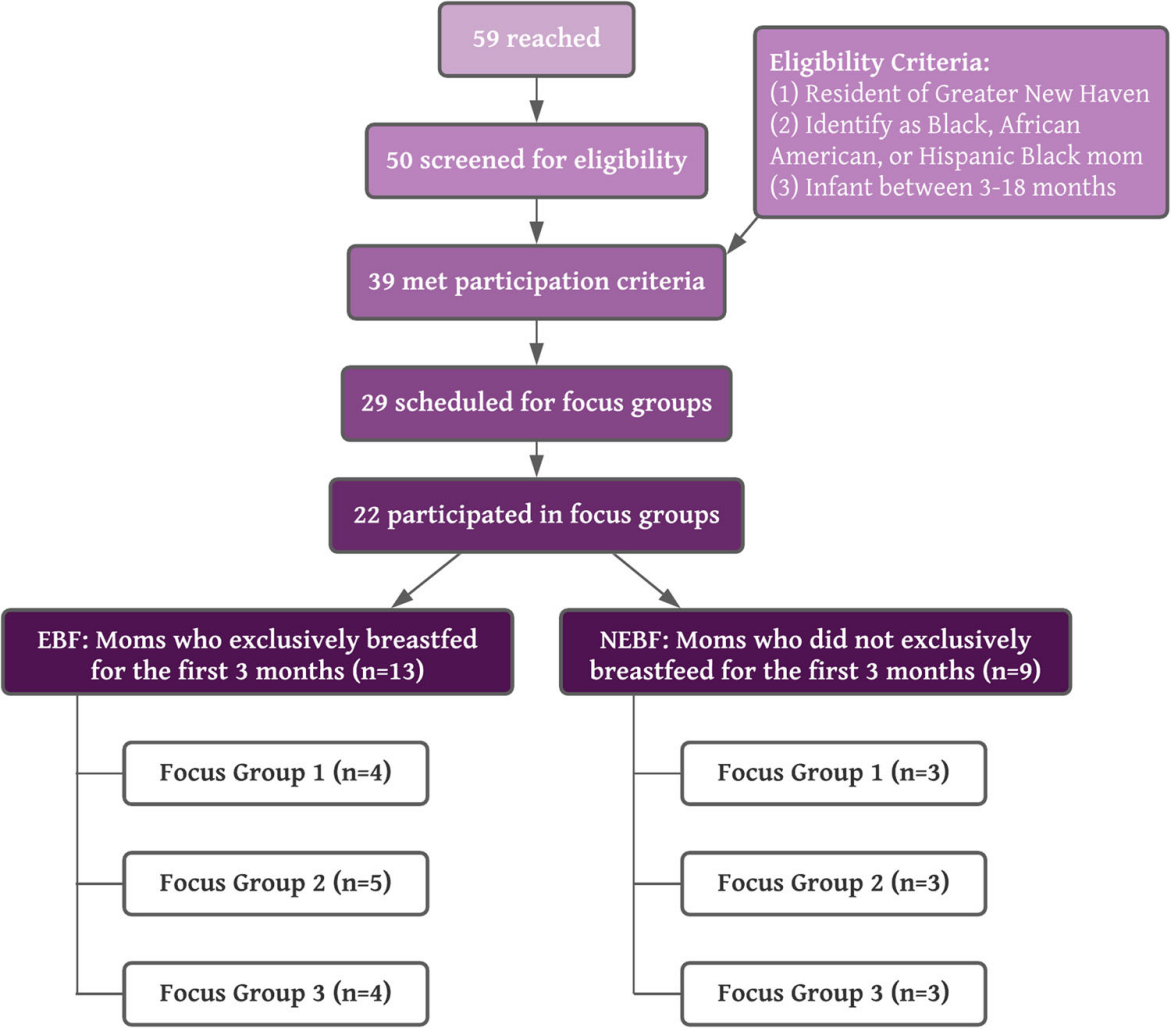
Participant characteristics

Recruitment was conducted through existing community partnerships, specifically with the support of New Haven Healthy Start. Focus groups were conducted from January 2021 through March 2021. All focus groups were conducted via Zoom due to physical distancing necessitated by the COVID‐19 pandemic. To ensure engagement from all participants, focus groups were intentionally small (three to six participants). As shown in Figure 1, a total of 22 individuals participated. There were six focus groups overall: three for EBFs (*n* = 13 participants) and three for NEBFs (*n* = 9 participants). A minimum of three focus groups each for the EBF and NEBF groups was chosen to allow for comparison and to assess newly emerging themes and ideas. Sessions averaged 90 minutes and were audio‐recorded. Recordings were transcribed, and transcriptions were deidentified and kept on a secure server. Participants were compensated with a $40 gift card. The study was approved by the Institutional Review Board of Yale University.

### 2.3 Data analysis

Deidentified transcripts were coded by a team of four researchers using Dedoose, with the first EBF and the first NEBF transcript coded by all four members. The research team used a combination of inductive and deductive coding, starting with a priori parent codes based on the theory‐derived determinants of the Barrier Analysis (see Table 1). The determinants of behaviour change formed the overarching themes that coders then inductively coded within, forming the child codes. Researchers independently coded the initial transcripts and then met to review codes and reach an agreement on the coding structure before coding the remaining transcripts. Sessions were held after each transcript was coded by at least two coders, and disagreements on codes or code applications were discussed until a consensus was reached. Codebook development and refinement took place after completing every transcript to adjudicate differences in code application.

**Table 1:** Parent Coding Structure, based on determinants of behaviour change in the Barrier Analysis Approach (Kittle, 2013)

	Determinant	Description
1.	Perceived self‐efficacy (what makes it easier to breastfeed)	An individual's belief that it is easier for them to do a particular behaviour given their current knowledge and skills. We ask: what makes it easier to perform the behaviour?
2.	Perceived self‐efficacy (what makes it harder to breastfeed)	An individual's belief that it is more difficult for them to do a particular behaviour given their current knowledge and skills. We ask: what makes it harder to perform the behaviour?
3.	Perceived positive consequences	What positive things a person thinks will happen as a result of performing a behaviour. Responses to questions related to positive consequences may reveal advantages (benefits) of the behaviour, attitudes about the behaviour and perceived positive attributes of the action
4.	Perceived negative consequences	The negative things a person thinks will happen as a result of performing a behaviour. Responses to questions related to negative consequences may reveal disadvantages of the behaviour, attitudes about the behaviour and perceived negative attributes of the action
5.	Perceived social norms (supportive people)	The perception that people important to the mother think that the mother should do the behaviour. Norms have two parts: who matters most to the mother on a particular issue and what the mother perceives those people think the mother should do.
6.	Perceived social norms (unsupportive people)	The perception that people important to the mother think that the mother should not do the behaviour. Norms have two parts: who matters most to the mother on a particular issue and what the mother perceives those people think the mother should do.
7.	Access	Includes the degree of availability (to a particular audience) of the needed products or services required to adopt a given behaviour. Includes barriers and facilitators related to race, cost, geography, distance, linguistics, cultural issues, gender or gender identity, etc.
8.	Perceived action efficacy	The belief that by practicing the behaviour (breastfeeding), one will avoid the problem that the behaviour is effective in avoiding (e.g., if I breastfeed I avoid my baby being susceptible to sickness). We ask: did the mother state that breastfeeding had an effect of X, Y, Z condition?
9.	Perceived divine will (influence of religious belief)	A person's belief that it is God's will for them to/not to breastfeed. Includes participant's perception of what their religion accepts or rejects and perceptions about the spirit world or magic (e.g., spells, curses)
10.	Policy	Laws and regulations (local, regional or national) that affect behaviours and access to products and services that may make it more or less likely for a person to take steps to breastfeed
11.	Culture	Cultural norms or stigmas that affect infant feeding or breastfeeding behaviour

The major barriers and facilitators to breastfeeding were then identified through a combination of two approaches: (1) identifying codes that were used frequently overall, and (2) identifying codes that differed between the EBF and the NEBF groups. Barriers faced only by those who did not breastfeed exclusively for at least 3 months, for example, may be more critical to address. Conversely, facilitators that emerged only among those who exclusively breastfed should be supported and made known to all who desire to breastfeed, as well as to decision‐makers and all who support those who desire to breastfeed. In determining which barriers and facilitators were most pressing to address, the research team included themes that came up frequently or were raised as important by participants. A minimum of three focus groups each for the EBF and NEBF groups were chosen for comparison purposes. During the third focus groups in each group, researchers noted that new themes were not emerging and theme saturation had been reached. Codes that were frequently mentioned by participants, or were highlighted as important, were then categorized within the Ecological Model for Health Promotion developed by McLeroy et al. (1988) that builds upon Bronfenbrenner's (1977) multilevel framework of ecological influence, also known as the Socioecological Model. The McLeroy et al. (1988) model expands Bronfenbrenner's model by including five levels of influence on health behaviour and behaviour change: intrapersonal/individual factors, interpersonal factors, institutional factors, community factors and public policy. The Socioecological Model is a common framework used to examine social and structural levels of influence on breastfeeding behaviours and design corresponding interventions. This model has been used in numerous breastfeeding‐related studies (Bueno‐Gutierrez & Chantry, 2015; Scott et al., 2020; Segura‐Pérez et al., 2021; Shipp et al., 2022; Snyder et al., 2021; Tomori et al., 2022; Vilar‐Compte et al., 2022).

## 3 RESULTS

Twenty‐two Black/African American women (EBF = 13 women, NEBF = 9 women) participated in six focus group discussions, with a range of three to six participants per focus group (Figure 1). All women were at least 18 years old and from the Greater New Haven area. The EBF and NEBF groups were comparable in terms of the average age of their youngest child (EBF = 6.2 months old, NEBF = 6.3 months old). Most of the participants, including those in the NEBF groups, had tried breastfeeding at least once, and many of them breastfed for 3 months or more, but not exclusively. Only two NEBF participants out of nine did not breastfeed at all; the remaining NEBF participants breastfed and supplemented with formula.

**Figure 2:**
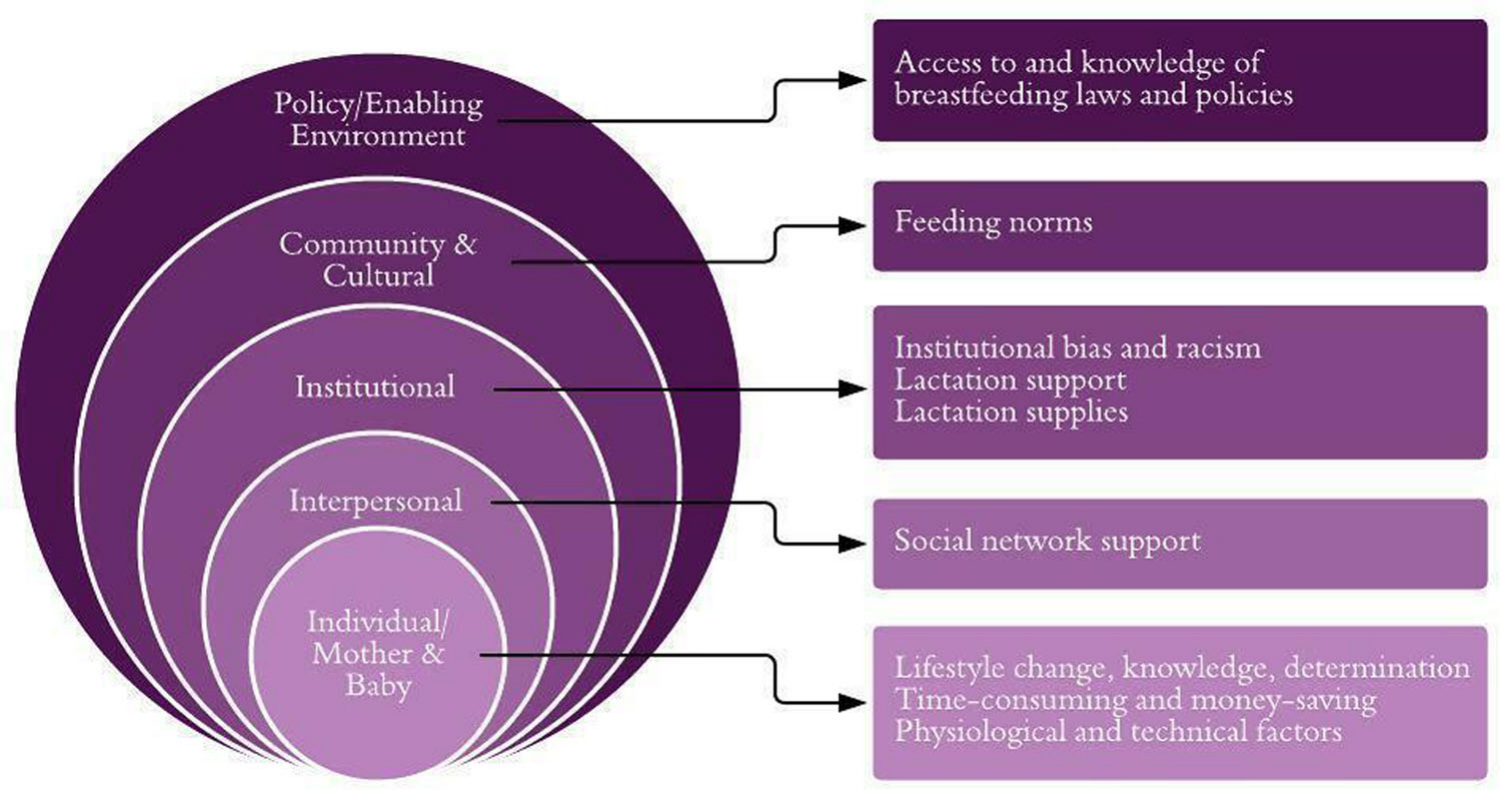
Focus group findings mapped onto the Socioecological Model

**Figure 3:**
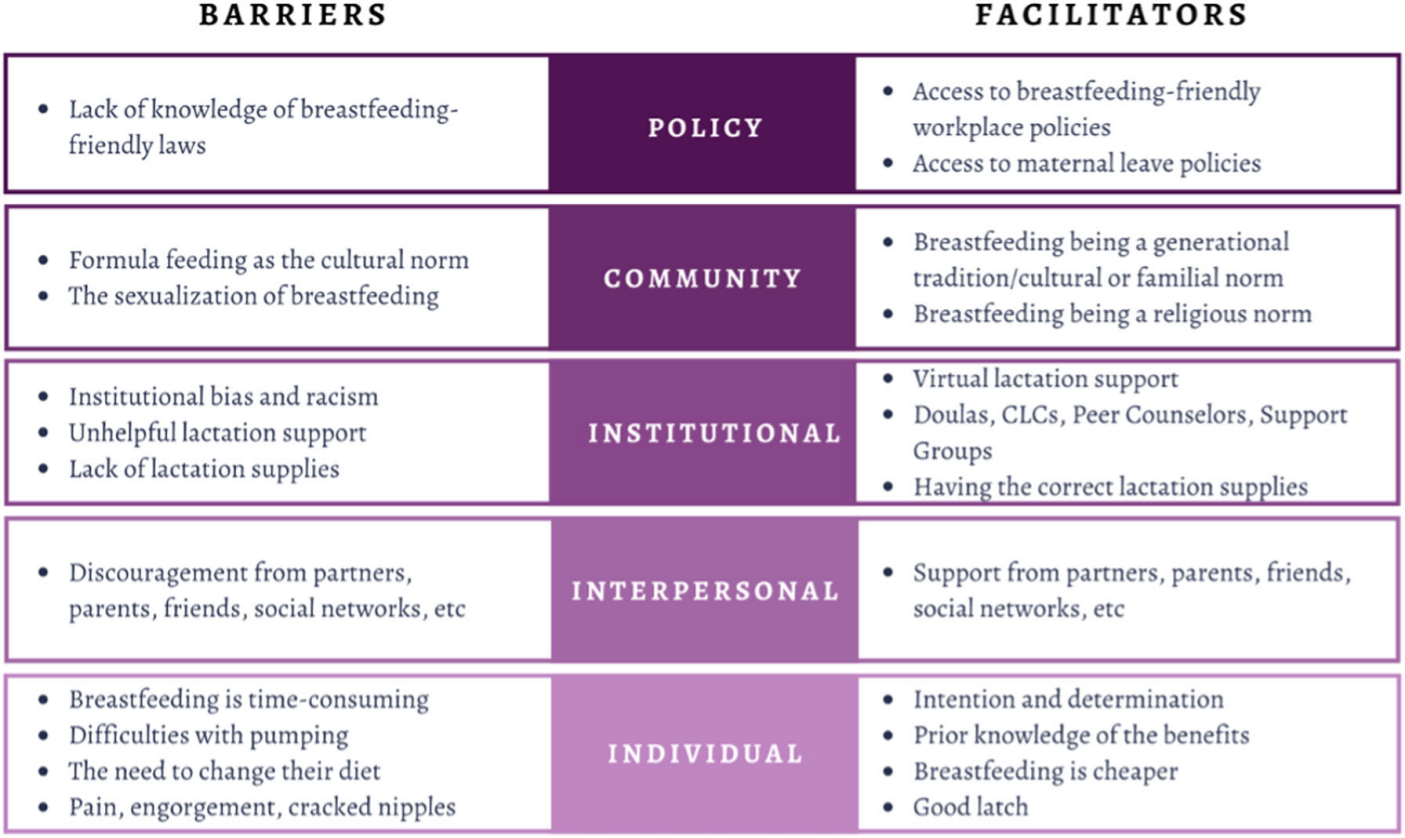
Barriers and facilitators to exclusive breastfeeding for up to 3 months

Emerging themes from both the EBF and NEBF groups were identified and grouped into levels of influence according to the Socioecological Model (Figure 2), and barriers and facilitators were further delineated (Figure 3). The following sections report focus group findings by the levels of the Socioecological Model.

### 3.1 Policy: Access to and knowledge of breastfeeding laws and policies

More than half of the participants (55%, *n* = 12) noted that there was little to no influence from any specific breastfeeding‐friendly policies in their decisions to breastfeed. In many instances, participants were unaware that breastfeeding‐friendly policies existed. Not knowing policies related to protections in the workplace posed a barrier to accessing and expressing their right to breastfeed.

Conversely, participants with knowledge of and access to breastfeeding‐friendly policies, particularly in the workplace (27%, *n* = 6), indicated that it was a facilitator to their ability to continue breastfeeding after returning to work. A private lactation space at work and access to maternity leave, or having a ‘place to go and time to do all of that’, were two breastfeeding‐friendly provisions in the workplace that some participants (18%, *n* = 4) mentioned as particularly helpful.

### 3.2 Community: Feeding norms

Some participants (27%, *n* = 6) shared that the cultural norm of sexualizing breasts and breastfeeding inhibits the practice of breastfeeding. Participants explained that ‘the stigma is still there’ around breasts having a sexual rather than a nurturing quality that ‘actually [symbolizes] what they're for’. Breast sexualization creates a stigma around breastfeeding and ‘[sexualizes] a child eating’, framing it as something that should not be done in public. Participants noted that if they wanted to breastfeed in public, they would need to bring a cloth to cover their breasts because people would tell them ‘you need to cover up’. Even then, they may receive ‘looks’ or comments on the inappropriateness of breastfeeding in public.

About a third of participants (36%, *n* = 8) also reported the perception that for many women of colour, formula feeding, rather than breastfeeding, is the default practice. This norm was perceived as a barrier to breastfeeding, as many women noted receiving pushback from their communities when trying to breastfeed. Additionally, several participants mentioned that ‘nobody [they knew] breastfed, or thought about it, or even tried’ to breastfeed and that their families thought they were ‘weird’ for wanting to try.

However, some participants (41%, *n* = 9) expressed having exposure to breastfeeding through family and described how ‘just hearing my mom say “I breastfed you” and my grandmother “I breastfed her”’ helped to make it a ‘tradition’ for them to breastfeed their babies as well. Similarly, participants who were surrounded by others who breastfed, such as those in religious communities where breastfeeding is the preferred practice and a ‘benefit for your children to be breastfed’ (18%, *n* = 4), found it to be an important facilitator and motivator to breastfeed.

### 3.3 Institutional: Lactation support

A barrier that about a third of participants (36%, *n* = 8) experienced was a lack of accessible and helpful lactation support. Participants stressed that the lack of lactation support outside of regular working hours, such as overnight, made it difficult to continue breastfeeding without receiving the needed help. Furthermore, if mothers were able to access lactation support, but the quality of the support was low, it was more of a barrier than a facilitator, especially if the nurse was not ‘well‐versed’ in how to set them up with breast pumps. Participants mentioned experiences with dismissive or inexperienced lactation support professionals as discouraging and unhelpful. They specifically discussed negative experiences with lactation support at the hospital and expressed that they felt the lactation specialists were ‘just making their rounds’ to ‘check you off’, regardless of if they actually provided support.

On the other hand, participants who received helpful and attentive lactation support that ‘walk[ed] you through’ the process found it to be a facilitator for breastfeeding. Among the types of lactation support specialists that the majority of participants (86%, *n* = 19) found most helpful were doulas, certified lactation counsellors and peer counsellors. Participants mentioned that having a lactation specialist who was reassuring and ‘explained a lot of it’ helped motivate them to keep trying with breastfeeding. Other types of lactation support that some participants (32%, *n* = 7) found helpful and wanted more access to were support groups and virtual/telehealth support.

### 3.4 Institutional: Lactation supplies

A few participants (18%, *n* = 4) noted that, given their situation, the lack of breastfeeding supplies or hardware, such as a breast pump, was a substantial barrier to being able to initiate breastfeeding. One NEBF participant discussed how a delay in paperwork led to a delay in the breast pump arriving (‘later than [it] was supposed to’), which impacted her ability to breastfeed. Without the breast pump, it ‘messed [her] up from the beginning’.

Consistent with the sentiment expressed by this participant, other participants (27%, *n* = 6) described having the correct lactation supplies as a facilitator to breastfeeding. They remarked on how the hospitals would set them up with breast pumps ahead of time and how they got ‘two different breast pumps from them’ so that they would have them ready for when they needed to switch to pumping.

### 3.5 Interpersonal: Social network support

The majority of participants (68%, *n* = 15) mentioned that discouragement or lack of support from their close social networks was a barrier. This discouragement came from their partners, mothers, in‐laws and friends. They felt pressure from those who were closest to them to ‘give that baby formula’ rather than breastfeed, which can make it more difficult for them to maintain breastfeeding.

Conversely, all participants (100%, *n* = 22) talked about how having the support of people around them and these people being ‘on board’ with their decision helped to facilitate breastfeeding. This support came from their partners, parents and friends. Types of support included emotional support, such as encouragement, as well as practical support, such as helping to feed the baby.

### 3.6 Individual: Lifestyle change, knowledge, determination

Some participants (36%, *n* = 8) said that the need to make a lifestyle or behaviour change made the transition into breastfeeding harder for them. One type of change that participants discussed as difficult was having to be on a specific maternal diet and needing to make sure they ‘[ate] the right food’. Restrictions on the types of food they should consume were a ‘disadvantage’. Another change mentioned as a barrier was having to abstain from alcohol, smoking or certain medications while breastfeeding.

An additional facilitator to breastfeeding that some participants (32%, *n* = 7) mentioned was having knowledge about the benefits and challenges of breastfeeding, as well as how breast milk is considered ‘liquid gold’ before actually engaging in breastfeeding. This knowledge that participants mentioned might have come from having previous personal experiences with breastfeeding, learning from the experiences of their friends and family, researching breastfeeding on their own or learning about its benefits from their health care providers. Similarly, having prior knowledge of the potential difficulties of breastfeeding helped them to anticipate any challenges they might have.

Most participants (72%, *n* = 16) also noted that having prior intention or determination to breastfeed helped them to manage their difficulties with breastfeeding. Participants talked about how they were determined to breastfeed ‘no matter what’ and how that determination helped to facilitate their breastfeeding. They discussed how their understanding of the benefits of breastfeeding helped to motivate them because they felt they were making the ‘best’ decision for their baby's health.

### 3.7 Individual: Time‐consuming and money‐saving

A barrier to breastfeeding that some participants (36%, *n* = 8) reported was how time‐consuming breastfeeding could be. This was especially true for participants who were pumping. They talked about how pumping can require advanced planning and setup, and how ‘intentional’ they had to be with their schedules since it ‘takes time to breastfeed’.

Other participants (32%, *n* = 7) communicated that breastfeeding helped them save money, which facilitated their decisions to breastfeed. They did not have to ‘go out and buy formula every week’, which can be prohibitively expensive. Therefore, breastfeeding was viewed as the less expensive option for infant feeding.

### 3.8 Individual: Physiological and technical factors

Half of all participants (50%, *n* = 11) described their experiences with breastfeeding as ‘painful’. The pain of engorgement, cracked nipples and sore breasts were barriers to continuing breastfeeding, as participants mentioned wanting to ‘give up’. Some participants stopped breastfeeding because the pain was too much.

Some participants (32%, *n* = 7) also mentioned difficulties with pumping. When participants switched to pumping, they expressed feeling like they ‘couldn't pump as much’ and feeling like they could not give their babies ‘enough’. They felt pumping was affecting their milk supply, so they would elect to supplement with formula. Additionally, making the transition from breastfeeding to pumping was difficult because they had to bottle train their infants, which presented its own challenges.

On the other hand, a few participants who were able to get a good latch (18%, *n* = 4) felt that the good latch was a facilitator to breastfeeding their infants. The participants talked about how helpful it would be to have the baby ‘latch on really quick’ and have that latch be ‘comfortable’. Participants who were unable to get a good latch also discussed how it would have helped to be able to have access to help with latching, underlining the importance of the baby latching correctly, as well as additional networks of support during the process of trying to breastfeed.

Both groups mentioned difficulties with pain as a barrier, and more NEBF participants mentioned pumping and latching as issues that inhibited their ability to exclusively breastfeed.

### 3.9 Discerning barriers and facilitators

Looking at results by whether the mothers breastfed beyond 3 months, more EBFs reported having the support of their mothers and partners, as well as having prior knowledge of breastfeeding, strong intentions to breastfeed before birth, and the perception of breastfeeding as money‐saving. The barriers that arose more often for those that did not breastfeed beyond 3 months included inaccessible lactation supplies and support, difficulties with pumping, latching issues and the perception of breastfeeding as time‐consuming. These distinguishing facilitators and barriers are noted in Figure 3.

### 3.10 Of note: Institutional bias and racism

One participant who was in the EBF group noted that she felt public health institutions and organisations only recently started paying attention to and encouraging Black women to breastfeed, despite knowing the benefits of breastfeeding long before this time. She discussed how Black women have historically been ignored and have not ‘[been] paid that much attention to’ in health promotion efforts, and how this ‘systemic’ bias could influence breastfeeding among Black women by excluding them. While participants at large did not call attention to racism or institutional bias directly, they spoke of situations where they were ignored, overlooked, dismissed or not given quality care. As Black women reported this, it is likely that these experiences were related to bias and racism. For this reason, the research team thought this theme was important to note.

## 4 DISCUSSION

This study adds to the evidence and builds an understanding of barriers that Black women face around exclusive breastfeeding across all levels of the Socioecological Model. We also add to the understanding of facilitators and community strengths that can support exclusive breastfeeding among Black mothers. At the policy level, a common barrier was a lack of knowledge about the policies or laws that protect the right to breastfeed, particularly in the workplace. As DeVane‐Johnson et al. (2017) point out in their research, breastfeeding‐friendly workplace policies, such as providing time and space to pump, can be conducive to continued breastfeeding because they allow mothers to continue expressing milk regularly, which is important for their milk supply. This is especially important, as continued employment or returning to work without adequate support or breaks for pumping is a noteworthy barrier to continued breastfeeding that is supported by both the literature (DeVane‐Johnson et al., 2017) and this study. By pointing out issues of unsupportive workplace policies and health care institution practices, we contribute to the growing literature on systemic bias in breastfeeding support for Black mothers (Robinson et al., 2019).

Within the community and cultural level, an important barrier that participants identified was the historical stigmatization and sexualization of breastfeeding. Previous studies have noted that such stigmatization and sexualization may be amplified for Black women, who have a history of being sexualized in popular media (Benard, 2016; Freeman, 2018, 2019; Johnson, 2018). Disentangling the sexualization of the breasts and the nurturing quality of breastfeeding is critical in fostering a cultural norm that is favourable for breastfeeding, especially in public. Participants also identified strong social bias as another barrier to exclusive breastfeeding. In fact, four mothers who were immigrants noted that there is a significant difference in how breastfeeding is viewed in their countries of origin compared to the United States, where it is still stigmatized. This is consistent with research that shows that breastfeeding rates for foreign‐born Black mothers in the United States are much higher than for US‐born Black mothers (Fabiyi et al., 2016; Safon et al., 2021). By describing sociocultural pressures and stigma, we add to the literature around social norms that can be transformed to increase breastfeeding rates (Carlin et al., 2019).

At the institutional level, participants noted that they did not feel heard or listened to when trying to receive support with breastfeeding, which is consistent with research that states that Black mothers are less likely to receive breastfeeding counselling from their providers (Beal et al., 2003; Kulka et al., 2011). One participant made a poignant remark about how she felt that Black women were previously ignored in any breastfeeding promotion efforts, despite the health benefits being well known. Research supports that it was not until the last couple of decades that Black women were included in breastfeeding promotion (Freeman, 2019).

On the interpersonal level, participants identified an overall lack of support from mothers' social networks and those who were closest to them. Previous research has shown that breastfeeding behaviour is influenced by a mother's social network and that just having one supportive person in their network can be enough to encourage breastfeeding (Carlin et al., 2019). This study builds upon this literature, highlighting the perceived importance of the role that mothers and partners of the breastfeeding woman play in her ability to exclusively breastfeed.

At the individual level, participants identified common barriers to exclusive breastfeeding as having to return to work and having to make significant lifestyle changes, such as changing diets or refraining from alcohol or cigarette use, while breastfeeding. These barriers are tantamount to those identified in other studies (Alexander et al., 2010; Hedberg, 2013; Ware et al., 2014). Furthermore, other activities that participants mentioned, such as addressing difficulties related to pumping and latching, increasing access to lactation support and supplies and sharing information on the health and financial benefits of breastfeeding with expectant moms, could be included in individual‐level interventions, while simultaneously addressing interpersonal, institutional, community and policy factors. These activities are also supported by prior research on barriers to breastfeeding and how to improve breastfeeding rates (Davis et al., 2021; Shipp et al., 2022; Tang et al., 2019).

The unique combination of these factors among individuals across the Socioecological Model impacts whether mothers are able to initiate breastfeeding, exclusively breastfeed and/or meet their breastfeeding intentions. There is not necessarily a single, isolated barrier that is the determining factor. Rather, these barriers operate altogether, creating a complex environment that can be either favourable or unfavourable to exclusive breastfeeding.

## 5 STRENGTHS AND LIMITATIONS OF STUDY DESIGN

### 5.1 Strengths

A strength of the Barrier Analysis design was that it provided a framework for a range of questions based on behaviour change theories, including the Health Belief Model, Social Cognitive Learning Theory and the Transtheoretical Model. It also provided a process for considering the strength of themes based on how often they were noted, and whether noted by EBFs or NEBFs. For example, if EBFs mentioned partners supporting breastfeeding as a facilitator but NEBFs did not, then partners' support may be an important component for facilitating exclusive breastfeeding behaviour. The Barrier Analysis design also provided a framework for dissemination and uptake of findings, including community‐level action planning. For example, after completing the analysis, our team used the Barrier Analysis Designing for Behaviour Change Framework (TOPS, 2017) to elicit community responses around key findings and support action planning in the New Haven community.

### 5.2 Limitations

While this study has provided rich and detailed descriptions of the experiences of Black mothers and their breastfeeding journeys, there were some important limitations to note. Even though the research team distributed the recruitment flyer through social media and community organisations, the majority of participants ended up being referred through one community partner organisation that has invested in promoting breastfeeding. Likely due to this, most of our participants, including those in the NEBF groups, had tried breastfeeding at least once, resulting in data that largely reflect those with at least some breastfeeding experience. Therefore, it was not surprising that EBF and NEBF participants reported several similar barriers and facilitators to breastfeeding. We may have seen more substantial differences between those who did and did not exclusively breastfeed for 3 months if the inclusion criteria for NEBF participants had been more stringent, only recruiting those who did not breastfeed at all.

Another limitation was the small sample size, although discussion of the same or similar themes across focus groups suggests we reached information saturation with this sample size. As the study occurred during the COVID‐19 pandemic, recruitment was challenging and was carried out primarily virtually or through community partners. As the Black community was particularly hard‐hit by the COVID‐19 pandemic in New Haven, it is likely that many mothers from the recruitment pool were prioritizing other concerns related to the pandemic. Further, it proved more difficult to recruit NEBF participants than EBF participants, and multiple NEBF focus group sessions had to be cancelled and rescheduled, suggesting there may have been some systematic differences in EBF and NEBF participants in terms of willingness to participate in a study about infant feeding. To address this, we used inclusive language in the flyer that emphasized that we were looking to recruit both mothers who breastfed and those who did not. However, as breastfeeding is an encouraged healthy behaviour, participants who formula‐fed may have felt the stigma around discussing their infant feeding choices in a group setting. Indeed, one NEBF participant mentioned that she ‘knows it [formula feeding] makes her look bad’, which suggests that other mothers may have felt similar shame around formula feeding and been hesitant to share their personal choices.

Additionally, although recruitment was open to all gender identities, this study only included mothers identifying as women. Future studies could use a similar approach to assess the barriers and facilitators to breast‐ or chestfeeding, intentionally recruiting multiple gender identities.

## 6 CONCLUSION

This study adds to the literature on Black mothers' experiences and perspectives around breastfeeding, highlighting key barriers and facilitators to exclusive breastfeeding during the first 3 months. This study has important implications for programme and policy design, for public health practitioners who seek to address racial disparities in breastfeeding rates, and for researchers designing studies around breastfeeding from a health equity lens (Rhodes et al., 2021). As demonstrated by the findings of this study, Black women face unique and often systemic barriers to breastfeeding at every level, including at the policy, cultural, institutional and interpersonal levels. In addition, this study highlights clear facilitators to exclusive breastfeeding, which may serve as a point of action for public health programme planning that centres on existing strengths in the Black community. Policy and local intervention efforts focused on addressing the identified barriers and facilitators may work to close the gap in breastfeeding rates, and the ability of women of colour to meet their breastfeeding intentions (Pérez‐Escamilla & Sellen, 2015). Study findings support the use of the Socioecological Model to design multicomponent interventions to improve breastfeeding outcomes in Black and African American women (Segura‐Pérez et al., 2021).

## AUTHOR CONTRIBUTIONS

Kathleen O'Connor Duffany was the Principal Investigator of this study. Victoria Tran, Amelia Reese Masterson, Tomeka Frieson and Kathleen O'Connor Duffany designed the research study. All authors revised protocols and guides. Victoria Tran, Amelia Reese Masterson, Tomeka Frieson and Frankie Douglass were involved in data collection, and Amelia Reese Masterson led study management. Victoria Tran, Amelia Reese Masterson, Tomeka Frieson and Frankie Douglass analysed the data. All authors reviewed and developed themes, results and implications. Victoria Tran wrote the first draft of the manuscript. All authors were engaged in manuscript development and provided substantive comments. All authors read and approved the final manuscript.

## CONFLICT OF INTEREST

The authors declare no conflict of interest.

## Data Availability

De‐identified data that support the findings of this study are available from the corresponding author upon request.
